# A comparison of eflapegrastim to pegfilgrastim in the management of chemotherapy‐induced neutropenia in patients with early‐stage breast cancer undergoing cytotoxic chemotherapy (RECOVER): A Phase 3 study

**DOI:** 10.1002/cam4.3227

**Published:** 2020-07-20

**Authors:** Patrick Wayne Cobb, Yong Wha Moon, Klára Mezei, István Láng, Gajanan Bhat, Shanta Chawla, Steven J. Hasal, Lee S. Schwartzberg

**Affiliations:** ^1^ St. Vincent's Frontier Cancer Center Frontier Cancer Center Billings MT USA; ^2^ CHA Bundang Medical Center Seongnam Korea; ^3^ Szabolcs‐Szatmár ‐ Bereg County Hospital Nyíregyháza Hungary; ^4^ National Institute of Oncology Budapest Hungary; ^5^ Spectrum Pharmaceuticals, Inc Irvine CA USA; ^6^ West Cancer Center Germantown TN USA

**Keywords:** chemotherapy‐induced neutropenia, eflapegrastim, ESBC, pegfilgrastim, Pegfilgrastim, Rolontis

## Abstract

Eflapegrastim (Rolontis^®^) is a novel, long‐acting hematopoietic growth factor consisting of a recombinant human granulocyte‐colony stimulating factor (rhG‐CSF) analog conjugated to a human IgG4 Fc fragment via a short polyethylene glycol linker. We report results from a second pivotal, randomized, open‐label, Phase 3 study comparing the efficacy and safety of eflapegrastim to pegfilgrastim for reducing the risk of chemotherapy‐induced neutropenia. Patients with Stage I to IIIA early‐stage breast cancer (ESBC) were randomized 1:1 to fixed‐dose eflapegrastim 13.2 mg (3.6 mg G‐CSF) or pegfilgrastim (6 mg G‐CSF) administered one day after standard docetaxel/cyclophosphamide (TC) therapy for four cycles. The primary objective was to demonstrate noninferiority (NI) of eflapegrastim compared to pegfilgrastim in mean duration of severe neutropenia (DSN; Grade 4) in Cycle 1. A total of 237 eligible patients were randomized 1:1 to receive either eflapegrastim (n = 118) or pegfilgrastim (n = 119). Cycle 1 severe neutropenia was observed in 20.3% (n = 24) of patients receiving eflapegrastim and 23.5% (n = 28) receiving pegfilgrastim. The DSN of eflapegrastim in Cycle 1 was noninferior to pegfilgrastim with a mean difference of −0.074 days (NI *P*‐value < .0001). Noninferiority was maintained throughout the four treatment cycles (*P* < .0001 in all cycles). Other efficacy endpoints results were comparable between treatment arms, and adverse events, irrespective of causality and grade, were comparable between treatment arms. The results demonstrate noninferior efficacy and comparable safety for eflapegrastim, at a lower G‐CSF dose, vs pegfilgrastim. The potential for the increased potency of eflapegrastim to deliver improved clinical benefit warrants further clinical study.

## Graphical abstract

This study demonstrates that eflapegrastim has noninferior efficacy to pegfilgrastim, at a lower G‐CSF dose, in all four cycles of TC treatment. Eflapegrastim had comparable safety to pegfilgrastim with no unexpected adverse events.

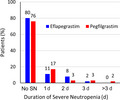

## 1 INTRODUCTION

Myelosuppression, particularly neutropenia, has presented a major challenge in cancer treatment since the introduction of cytotoxic chemotherapy in the 1950s. Availability of the first recombinant human granulocyte‐colony stimulating factor (rhG‐CSF) in the 1990s (filgrastim) provided a safe and effective means to reduce the considerable burden of infection‐related morbidity and mortality associated with chemotherapy‐induced neutropenia (CIN).^1^

The advent of pegfilgrastim, the first long‐acting rhG‐CSF, a decade later simplified supportive care for CIN with a once‐per‐chemotherapy‐cycle option.^2^ Since then, supportive care options for CIN have not changed other than through the introduction of biosimilar alternatives.^3^

Eflapegrastim (Rolontis^®^, SPI‐2012, HM10460A) is a nonbiosimilar, long‐acting hematopoietic growth factor that represents the first myeloid growth factor innovation in more than 15 years. The eflapegrastim molecule (72 kDa) consists of an rhG‐CSF analog (17th 65th Ser‐G‐CSF, no additional N‐terminal Met) and a recombinant human IgG Fc fragment conjugated at their N‐termini via a short (3.4 kDa) polyethylene glycol linker. The strategy of adding an Fc fragment to extend drug half‐life has been used in marketed biologics (eg, etanercept, aflibercept, dulaglutide) that have been safely and effectively administered to hundreds of thousands of patients.^4^ Eflapegrastim shows increased uptake to the bone marrow, presumably due to the interaction of its Fc fragment with Fc receptors (FcRn) on the vascular endothelial surface.^5^ The resulting increased potency, as demonstrated by pharmacokinetic and pharmacodynamic data from preclinical and Phase 1 and 2 studies,^5^, ^6^, ^7^ gives eflapegrastim the potential to have an improved therapeutic index compared to pegfilgrastim. In nonclinical primate and rat studies, eflapegrastim demonstrated greater biologic activity than pegfilgrastim at reducing neutropenia, at one‐third the G‐CSF dose of pegfilgrastim.^8^, ^9^

Here we report the results of a second pivotal Phase 3 randomized study comparing eflapegrastim to pegfilgrastim (RECOVER, NCT02953340) in patients with Stage I to IIIA early‐stage breast cancer (ESBC) undergoing standard docetaxel/cyclophosphamide (TC) therapy, which demonstrates confirmatory, reproducible evidence from another nearly identically designed, independent study (ADVANCE, NCT02643420) conducted in the same patient population. In contrast to Phase 1 and 2 eflapegrastim studies using weight‐based dosing,^6^, ^7^ this Phase 3 study tested a fixed dose of 13.2 mg eflapegrastim (3.6 mg G‐CSF) that is equivalent to 60% of the 6 mg G‐CSF in pegfilgrastim. The 13.2 mg dose is equivalent to 188 µg/kg eflapegrastim (51 µg/kg G‐CSF) for a 70 kg person and was chosen based on the results of a Phase 2 dose‐ranging study, which showed noninferiority of eflapegrastim vs pegfilgrastim in the primary endpoint, mean Cycle 1 duration of severe neutropenia (DSN), for eflapegrastim 135 µg/kg (37 µg/kg G‐CSF) (0.44 vs. 0.31 days, *P* = .002), and statistical superiority at 270 µg/kg (74 µg/kg G‐CSF) (0.03 vs. 0.31 days, *P* = .023).^7^

## 2 MATERIALS AND METHODS

### 2.1 Participants and study design

Patients had Stage I to IIIA ESBC and were candidates for adjuvant or neoadjuvant TC therapy.^10^, ^11^ Key inclusion criteria included age ≥18 years, Eastern Cooperative Oncology Group (ECOG) performance status ≤2, adequate bone marrow function before the start of chemotherapy (absolute neutrophil count [ANC] ≥1.5 × 10^9^/L, platelets ≥100 × 10^9^/L, hemoglobin >9 g/dL), and adequate renal function (calculated creatinine clearance >50 mL/min and hepatic function (total bilirubin ≤1.5 mg/dL, aspartate aminotransferase (AST) and/or alanine aminotransferase (ALT) ≤2.5 × ULN, and alkaline phosphatase ≤2.0 × ULN). Exclusion criteria included known sensitivity to E coli‐derived products, L‐asparaginase, somatropin growth hormone, or recombinant interferon α‐2b; active infection, or ongoing treatment with antiinfectives, prior bone marrow or stem cell transplant, major surgery within 30 days prior to enrollment, or any other malignancy within 5 years prior to enrollment. All patients provided written informed consent and the study protocol was approved by Institutional Review Boards and/or Ethics Committees at all sites.

Eligible patients were randomized 1:1 to receive a single, fixed dose of eflapegrastim 13.2 mg (3.6 mg G‐CSF) or pegfilgrastim (6 mg G‐CSF) by subcutaneous injection on Day 2 of each cycle (~24 hours postchemotherapy).

Patients received up to four cycles of standard TC (docetaxel 75 mg/m^2^/cyclophosphamide 600 mg/m^2^), given by IV infusion on Day 1 of each cycle and dose modifications were not permitted in Cycle 1. Dose modifications for eflapegrastim or pegfilgrastim were not permitted.

Blood samples for complete blood counts (CBCs) with differential were collected pretreatment and on Day 1 and daily on Days 4‐15 of Cycle 1, and on Days 1, 4, 7, and 15 in subsequent cycles. However, if an ANC ≤1.0 × 10^9^/L was reported at any time in Cycles 2‐4, daily CBCs were performed until the ANC recovered to ≥1.5 × 10^9^/L. All blood analyses were performed at an independent central laboratory.

Patients were monitored for adverse events (AEs) for the duration of the study and serum chemistry was collected in every cycle. AEs and laboratory values were graded according to NCI CTCAE version 4.03. Safety assessments began with the first dose of TC and lasted until 35 (±5) days after the last dose of eflapegrastim/pegfilgrastim. To assess immunogenicity, blood samples were collected on Day 1 of each cycle, at the end‐of‐treatment visit, and during the long‐term follow‐up visits at 6 and 12 months. All immunogenicity tests were performed at independent laboratories.

### 2.2 Clinical endpoints

The primary efficacy endpoint was the duration of severe neutropenia (DSN) in Cycle 1, defined as the number of days of severe neutropenia (ANC <0.5 × 10^9^/L; Grade 4 per NCI CTCAE, v 4.03) from the day of first occurrence of an ANC below that threshold. In addition to DSN in Cycles 2‐4, other secondary endpoints assessed in each cycle included time‐to‐ANC recovery (time‐from‐chemotherapy administration to ANC ≥1.5 × 10^9^/L after the expected nadir); depth of ANC nadir (lowest ANC value); incidence of febrile neutropenia (FN; ANC <1.0 × 10^9^/L and either temperature >38.3°C or two consecutive readings ≥38.0°C over 2 hours); incidence of neutropenic complications (antiinfective use and/or hospitalizations); relative dose intensity (RDI); and safety.

### 2.3 Statistical analysis

This open‐label, multicenter, active‐control study was designed as one of two well‐controlled Phase 3 registration studies of eflapegrastim. The standard deviation results observed in previous Phase 3 pegfilgrastim studies were in the range of 1.4 and 1.5 days.^12^, ^13^ A sample size of 218 patients (109 in each arm) provides 90%, 86%, and 81% power to detect noninferiority using a one‐sided, two‐sample *t*‐test at a 2.5% level of significance, when the pooled standard deviation (SD) of the DSN is 1.4, 1.5, or 1.6 days, respectively.

All randomized patients were included in the intent‐to‐treat efficacy analysis. The safety population included all patients who received at least one dose of any study drug. The primary efficacy analysis compared the mean DSNs in Cycle 1 between the treatment arms based on a prespecified test of noninferiority hypothesis. A 2‐sided 95% confidence interval (CI) of the difference between the mean DSN of the two arms was calculated using a bootstrap resampling method, with treatment as the only stratification factor; the same method was used to assess mean DSNs in Cycles 2‐4 (95% CIs for other secondary endpoints were calculated using standard methods). Eflapegrastim was to be considered noninferior to pegfilgrastim if the upper limit of the two‐sided 95% CI for the difference in mean DSN was <0.62 days. This margin, based on the treatment effect observed in the pegfilgrastim pivotal studies,^12^, ^13^ eliminates the potential for biocreep when establishing noninferiority of two long‐acting G‐CSFs. Descriptive statistics were used to summarize patient disposition, patient demographics, and safety. Univariate and multivariate analyses were conducted post hoc to explore potential treatment effects for eflapegrastim vs. pegfilgrastim for patient subgroups, by age, weight, and other demographic characteristics.

## 3 RESULTS

### 3.1 Patient characteristics

**FIGURE 1:**
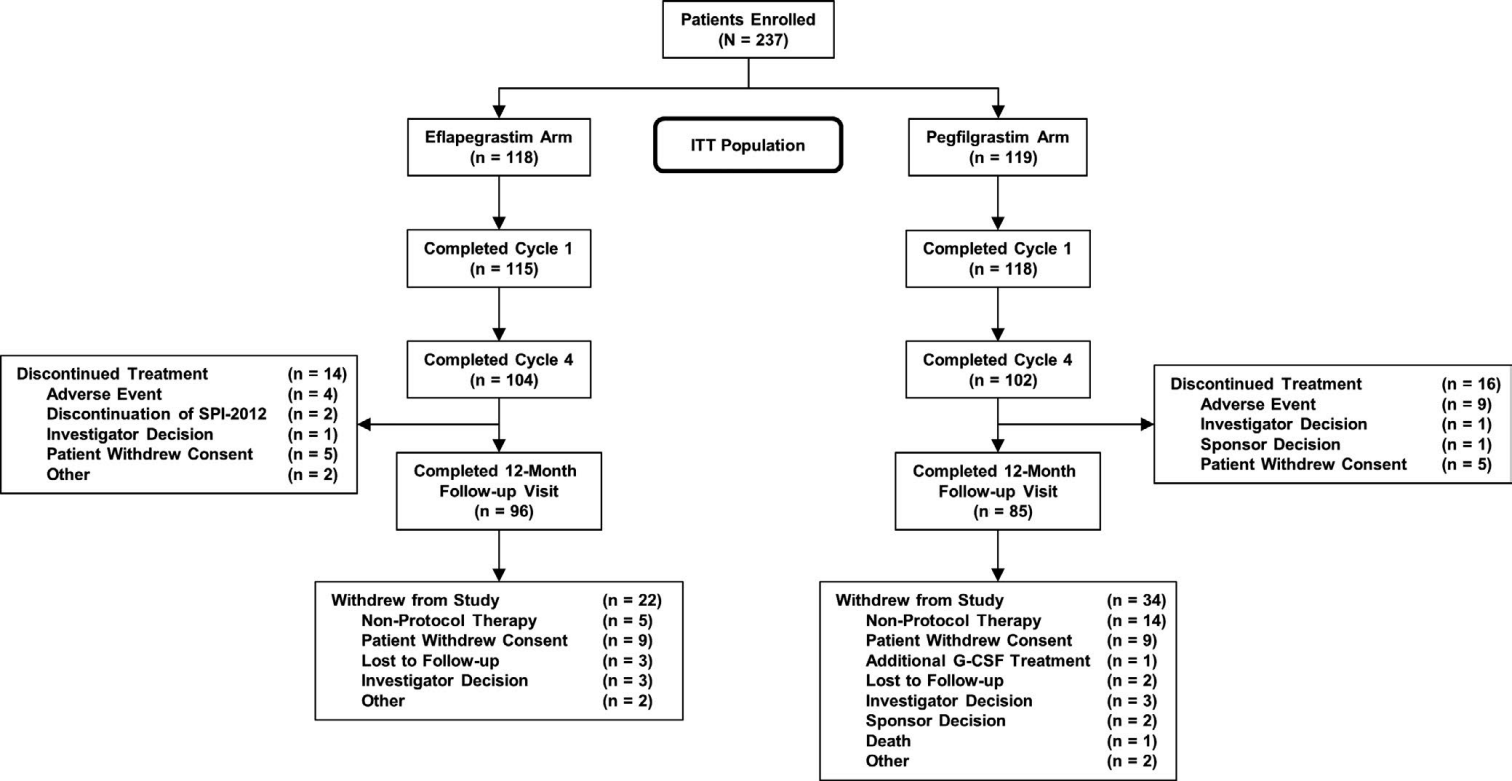
Patient disposition

A total of 237 patients (118 in the Eflapegrastim Arm, 119 in the Pegfilgrastim Arm) were enrolled (Figure 1) between July 2017 and May 2019 at 74 study sites, primarily within the United States (77%), with other sites in Canada (two sites), Hungary (six sites), Poland (seven sites), India (two sites), and Korea (eight sites). Two patients were included in the intent‐to‐treat (ITT) analysis but excluded from the safety analysis as they did not receive any protocol‐specified study drug (one patient in each arm). Ultimately, the safety population incorporated 117 patients in the Eflapegrastim Arm and 118 patients in the Pegfilgrastim Arm.

The patient demographics and clinical disease characteristics in the two arms were similar (Table 1). The median patient age for the Eflapegrastim Arm was 58 years (range, 29‐80) and 59 years (range, 34‐88) for the Pegfilgrastim Arm. Most patients were treated with chemotherapy in the adjuvant setting (84% in the Eflapegrastim Arm, 77% in the Pegfilgrastim Arm) and had an ECOG performance status of 0 (84% in the Eflapegrastim Arm, 76% in the Pegfilgrastim Arm). The median patient weight at baseline was 74.7 kg (range, 40.3‐171.4) in the Eflapegrastim Arm and 74 kg (range, 46.0‐152.8) in the Pegfilgrastim Arm; nearly 50% of patients in each treatment arm weighed more than 75 kg.

**TABLE 1:** Patient demographics and baseline characteristics

Characteristic	Eflapegrastim n = 118	Pegfilgrastim n = 119
Age, years		
Median (range)	58 (29‐80)	59 (34‐88)
<65 years, n (%)	74 (63)	79 (66)
≥65 years, n (%)	44 (37)	40 (34)
Weight, kg		
Mean (SD)	77.2 (20.05)	76.6 (20.36)
Median (range)	74.7 (40.3, 171.4)	74.0 (46.0, 152.8)
Weight group, n (%)		
<65 kg	32 (27)	36 (30)
65‐75 kg	28 (24)	27 (23)
>75 kg	58 (49)	56 (47)
Gender, n (%)		
Female	118 (100)	119 (100)
Race, n (%)		
White	85 (72)	96 (81)
Black	11 (9)	7 (6)
Other	22 (19)	16 (13)
ECOG performance status, n (%)		
0	99 (84)	90 (76)
1	19 (16)	27 (23)
2	0 (0)	2 (2)
Stage at diagnosis, n (%)		
Stage I	36 (31)	36 (30)
Stage IIA	40 (34)	46 (39)
Stage IIB	28 (24)	29 (24)
Stage IIIA	14 (12)	8 (7)
Histology type, n (%)		
Ductal Invasive	91 (77)	98 (82)
Ductal other	0 (0)	2 (2)
Lobular invasive	17 (14)	6 (5)
Mixed	1 (1)	3 (3)
Other	9 (8)	10 (8)
Treatment setting, n (%)		
Adjuvant	99 (84)	92 (77)
Neo‐adjuvant	19 (16)	27 (23)

Note

Abbreviation: ECOG, Eastern Cooperative Oncology Group.

### 3.2 Severe neutropenia

**FIGURE 2:**
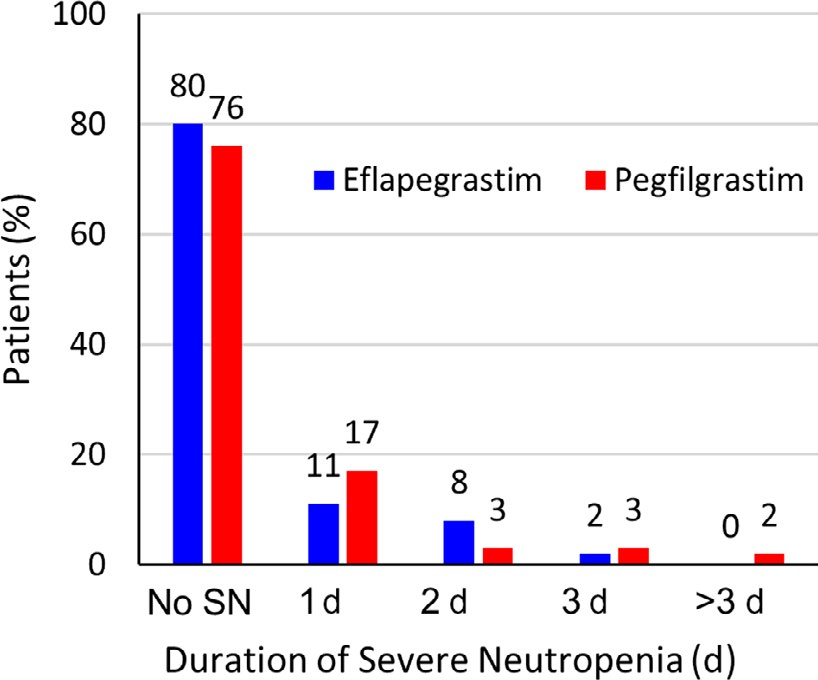
Duration of severe neutropenia (SN) in Cycle 1 (ANC <0.5 × 10^9^/L; Grade 4 per NCI CTCAE, V 4.03) for fixed dose 13.2 mg eflapegrastim (3.6 mg G‐CSF) and pegfilgrastim (6.0 mg G‐CSF)

The incidence of severe neutropenia (Grade 4, <0.5 × 10^9^/L) in Cycle 1 was 20.3% (n = 24) for the Eflapegrastim Arm and 23.5% (n = 28) for the Pegfilgrastim Arm. In the Eflapegrastim Arm, the DSN in Cycle 1 was 1 day in 13 (11%) patients, 2 days in 9 (8%) patients, and 3 days in 2 (2%) patients and in the Pegfilgrastim Arm, the DSN in Cycle 1 was 1 day in 20 (17%) patients, 2 days in 3 (3%) patients, 3 days in 3 (3%) patients, 4 days in 1 (1%) patient, and 7 days in 1 (1%) patient (Figure 2). Moreover, both drugs provided high levels of protection in Cycles 2‐4, with ≤8% of patient experiencing severe neutropenia in each cycle.

The mean (±SD) Cycle 1 DSN was 0.31 days (±0.69) for the Eflapegrastim Arm vs 0.39 days (±0.95) for the Pegfilgrastim Arm. The difference in mean DSN of −0.074 days (95% CI: −0.292, 0.129) between the two arms met the study's primary endpoint of noninferiority (*P* < .0001). The noninferiority in mean DSN between eflapegrastim and pegfilgrastim was maintained in Cycles 2‐4 (*P* < .0001, all cycles). The difference in the mean DSN in Cycle 2 was −0.016 days (95% CI: −0.117, 0.068), in Cycle 3 was 0.000 days (95% CI: −0.067, 0.068), and in Cycle 3 was −0.008 days (95% CI: −0.075, 0.060) (Table 2).

**TABLE 2:** Duration of severe neutropenia (ANC <0.5 × 109/L) in cycles 1 to 4

Cycle	Mean DSN, days (SD)		Difference (95% CI)	*P‐*value for Noninferiority
	Eflapegrastim n = 118	Pegfilgrastim n = 119		
Cycle 1^a^	0.31 (0.688)	0.39 (0.949)	−0.074 (−0.292, 0.129)	<.0001
Cycle 2	0.08 (0.267)	0.09 (0.432)	−0.016 (−0.117, 0.068)	<.0001
Cycle 3	0.07 (0.252)	0.07 (0.283)	0.000 (−0.067, 0.068)	<.0001
Cycle 4	0.07 (0.252)	0.08 (0.266)	−0.008 (−0.075, 0.060)	<.0001

Note

Abbreviations: CI, confidence interval; DSN, duration of severe neutropenia; SD, standard deviation.

^a^ Study primary endpoint.

The study was not prespecified or powered to confirm treatment effects in patient subgroups individually, although exploratory univariate subgroup analyses involving Cycle 1 DSN for age, race, treatment type (adjuvant/neoadjuvant), geographical region, and body weight demonstrated noninferiority between eflapegrastim and pegfilgrastim. Of special note, in patients ≥65 years of age, 44 patients in the Eflapegrastim Arm and 40 patients in the Pegfilgrastim Arm, the mean DSN for eflapegrastim (0.48 days) was noninferior to pegfilgrastim (0.50 days) with a difference of −0.023 (95% CI −0.397, 0.352). Furthermore, multivariate analyses of subgroups did not show any stratification effect for the difference in mean DSN (Table 3).

**TABLE 3:** Subgroup analysis of cycle 1 by subgroups

Subgroup	Eflapegrastim n = 118		Pegfilgrastim n = 119		Difference (95% CI^a^)
	n	Mean DSN (SD)	n	Mean DSN (SD)	
Age, years					
<65	74	0.22 (0.580)	79	0.33 (0.970)	−0.113 (−0.370, 0.145)
≥65	44	0.48 (0.821)	40	0.50 (0.906)	−0.023 (−0.397, 0.352)
Race					
White	85	0.32 (0.711)	96	0.36 (0.953)	−0.047 (−0.296, 0.202)
Non‐white	33	0.30 (0.637)	23	0.48 (0.947)	−0.175 (−0.599, 0.249)
Treatment setting					
Adjuvant	99	0.31 (0.695)	92	0.41 (1.007)	−0.100 (−0.345, 0.146)
Neoadjuvant	19	0.32 (0.671)	27	0.30 (0.724)	0.019 (−0.405, 0.444)
Region					
US	63	0.44 (0.819)	68	0.50 (1.113)	−0.056 (−0.396, 0.284)
Non‐US	55	0.16 (0.462)	51	0.24 (0.651)	−0.072 (−0.288, 0.145)
Weight, kg					
<65	32	0.38 (0.793)	36	0.31 (0.749)	0.069 (−0.304, 0.443)
65‐75	28	0.25 (0.518)	27	0.15 (0.362)	0.102 (−0.141, 0.344)
>75	58	0.31 (0.706)	56	0.55 (1.205)	−0.243 (−0.608, 0.122)

Note

Abbreviations: CI, confidence interval; DSN, duration of severe neutropenia; SD, standard deviation.

^a^ Two‐sided 95% CIs based on normal distribution.

### 3.3 ANC recovery

**FIGURE 3:**
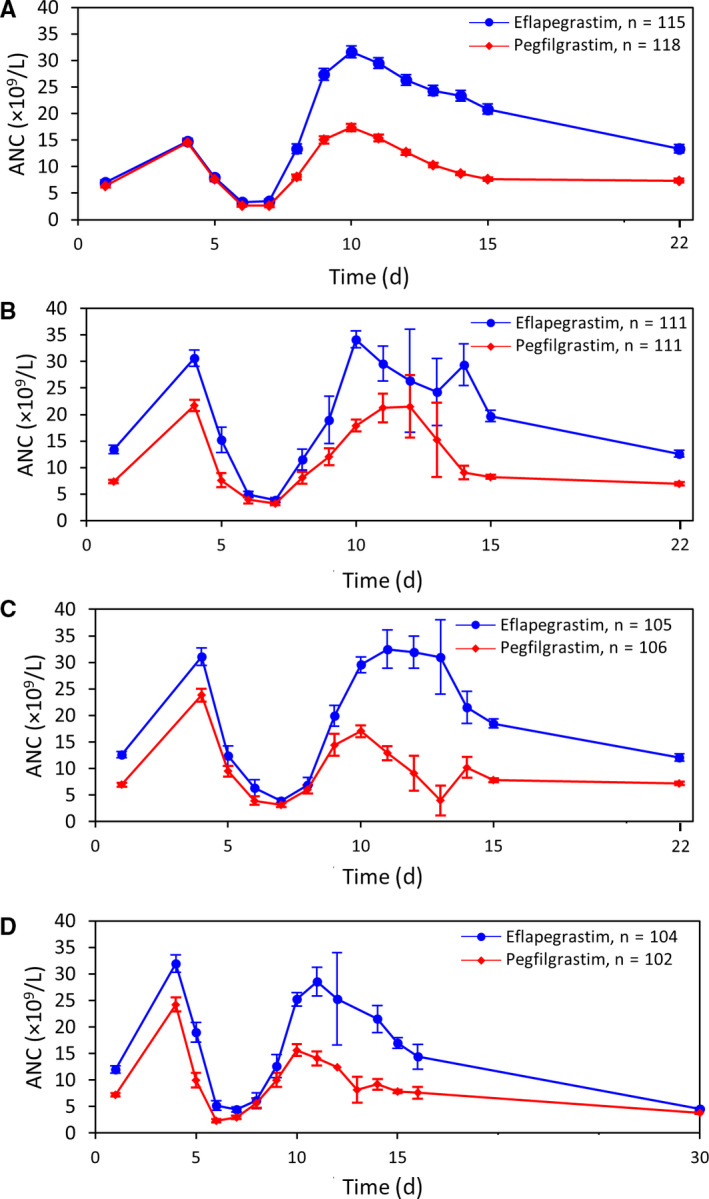
Mean (±SE) absolute neutrophil count (ANC) profiles for all patients administered either fixed dose 13.2 mg eflapegrastim (3.6 mg G‐CSF) or pegfilgrastim (6 mg G‐CSF) in Cycles 1‐4. (A) Cycle 1, (B) Cycle 2, (C) Cycle 3, (D) Cycle 4

The time‐to‐ANC recovery from chemotherapy administration on Day 1 to recovery to ≥1.5 × 10^9^/L was comparable between eflapegrastim and pegfilgrastim throughout all cycles (Figure 3). In each cycle, the ANC recovery peak for eflapegrastim was slightly greater than that for pegfilgrastim and these values were consistent across all four cycles. The ANC values at the end of each cycle remained higher for eflapegrastim than for pegfilgrastim and nearly returned to the base level before the start of the next cycle. At the end‐of‐study visit (30 days after the last G‐CSF dose), ANC counts were within normal limits.

The mean (±SD) time‐to‐ANC recovery in Cycle 1 was 3.49 days (±3.723) for eflapegrastim and 3.35 days (±3.745) for pegfilgrastim (*P* = .866), with similar but shorter recoveries in Cycles 2‐4 (Table 4). A further analysis of patients who experienced severe neutropenia (24 and 28 patients overall for the Eflapegrastim and Pegfilgrastim Arms, respectively) demonstrated comparable time‐to‐ANC recovery from nadir to 1.5 × 10^9^/L (1.38 days for eflapegrastim and 1.79 days for pegfilgrastim (*P* = .238). Median Cycle 1 ANC nadirs were 1.60 × 10^9^/L and 1.57 × 10^9^/L for eflapegrastim and pegfilgrastim, respectively (*P* = .363). The ANC nadir values in Cycles 2‐4 were higher in both treatment arms (Table 4).

**TABLE 4:** Secondary endpoints in cycles 1 to 4

Endpoint	Chemotherapy cycle							
	1		2		3		4	
	Efla	Peg	Efla	Peg	Efla	Peg	Efla	Peg
Time‐to‐ANC recovery								
Mean, days	3.49	3.35	2.19	1.96	1.96	2.08	1.93	1.67
*P*‐value	.87		.81		.89		.77	
Depth of ANC Nadir								
Median (×10^9^/L)	1.60	1.57	3.97	2.84	3.48	3.07	3.72	2.86
*P*‐value	.36		.14		.42		.52	
Incidence of febrile neutropenia								
n (%)	1 (0.8)	4 (3.4)	0 (0)	2 (1.7)	0 (0)	0 (0)	0 (0)	0 (0)
*P*‐value	.37		.50					
Incidence of neutropenic complications								
n (%)	1 (0.8)	5 (4.2)	1 (0.8)	1 (0.8)	0 (0)	1 (0.8)	1 (0.8)	0 (0)
*P*‐value	.21		.00		.00		.50	

Note

Abbreviations: ANC, absolute neutrophil count; Efla, eflapegrastim; Peg, pegfilgrastim.

### 3.4 Febrile neutropenia

The incidence of FN across all cycles was low for both treatment arms (Table 4), with one (0.8%) patient in the Eflapegrastim Arm and four (3.4%) patients in the Pegfilgrastim Arm developing FN in Cycle 1 (*P* = .370). During all treatment cycles, eflapegrastim was associated with fewer patients experiencing FN than pegfilgrastim (one patient vs. six patients, respectively), although the difference was not significant (*P* = .119). Since this is a comparative study with a standard of care treatment, the FN endpoint was not statistically powered to make any inference.

During Cycle 1, there was one (0.8%) patient in the Eflapegrastim Arm and five (4.2%) patients in the Pegfilgrastim Arm who experienced neutropenic complications (*P* = .213), and there were fewer patients in the Eflapegrastim Arm (n = 3; 2.5%) than in the Pegfilgrastim Arm (n = 7; 5.9%) who developed neutropenic complications over the four treatment cycles (*P* = .333). All patients with neutropenic complications, irrespective of treatment arm, were treated with anti‐infectives. The mean relative dose intensity (RDI) of docetaxel and cyclophosphamide in Cycle 1 was ≥99% in both arms and all but three (3%) patients in the Eflapegrastim Arm and all patients in the Pegfilgrastim Arm received 80% to 120% of the prescribed dose of docetaxel and all but one (1%) patient in the Eflapegrastim Arm and two (2%) patients in the Pegfilgrastim Arm received 80% to 120% of the prescribed dose of cyclophosphamide.

### 3.5 Safety analysis

Overall, the AEs observed in this study were consistent with those previously reported for patients receiving TC and other myeloid growth factors. Most patients experienced at least one treatment‐emergent AE (98% in both treatment arms), and most of these AEs were attributed to TC therapy with ≥90% of patients in both arms having AEs related to docetaxel and/or cyclophosphamide. The most common treatment‐emergent AEs (≥40% in either arm) related to either docetaxel or cyclophosphamide were lymphopenia, neutropenia, and nausea. All‐grade study drug‐related AEs (related to either eflapegrastim or pegfilgrastim) were reported in 63% of patients in the Eflapegrastim Arm and 61% in the Pegfilgrastim Arm. The most commonly observed study drug‐related AE was bone pain, reported in 40 (34%) patients (all grades) in the Eflapegrastim Arm and 45 (38%) patients in the Pegfilgrastim Arm (Table 5); Grade 3 bone pain was reported in two patients in the Eflapegrastim Arm and one patient in the Pegfilgrastim Arm. All Grade 3 bone pain was resolved with the use of analgesics. Other commonly reported study drug‐related AEs in both arms included arthralgia, back pain, and myalgia.

**TABLE 5:** Adverse events related to fixed dose 13.2 mg eflapegrastim (3.6 mg G‐CSF) or pegfilgrastim (6.0 mg G‐CSF) occurring in ≥ 5% of patients

	Eflapegrastim		Pegfilgrastim	
	n = 117		n = 118	
	n (%)		n (%)	
	Any grade	Grade 3/4	Any grade	Grade 3/4
Any event	74 (63)	16 (14)	72 (61)	8 (7)
Bone pain	40 (34)	2 (2)	45 (38)	1 (1)
Myalgia	17 (15)	2 (2)	11 (9)	0
Diarrhea	12 (10)	2 (2)	0	0
Back pain	11 (9)	2 (2)	5 (4)	1 (1)
Pyrexia	10 (9)	0	9 (8)	1 (1)
Arthralgia	9 (8)	1 (1)	7 (6)	1 (1)
Nausea	9 (8)	0	3 (3)	0
WBC count increased	9 (8)	4^a^ (3)	3 (3)	1^a^ (1)
Headache	8 (7)	1 (1)	7 (6)	1 (1)
Fatigue	7 (6)	0	10 (8)	1 (1)
Pain in extremity	7 (6)	0	4 (3)	0
Lymphocyte count decreased/lymphopenia	2 (2)	2 (2)	6 (5)	6 (5)

^a^ Patients’ WBC values were <100 × 10^9^/L, the criterion required for CTCAE version 4.03 Grade 3 WBC increased.

The incidence of AEs of special interest related to G‐CSF (musculoskeletal AEs, injection site reactions, and hypersensitivity‐type events) was comparable between the treatment arms, regardless of grade. Grade 4 study drug‐related lymphopenia was reported in one patient in the Eflapegrastim Arm and Grade 4 neutropenia and lymphopenia were reported in one patient in the Pegfilgrastim Arm. These Grade 4 study drug‐related AEs resolved. No leukocytosis (WBC >100 × 10^9^/L), splenic rupture, or anaphylaxis were reported in either treatment arm.

The incidence of serious AEs was lower in the Eflapegrastim Arm (10%) than in the Pegfilgrastim Arm (16%), the incidence of study drug‐related SAEs in the two arms was the same (2% in each arm). One patient in the Pegfilgrastim Arm died during Cycle 4 due to chronic obstructive pulmonary disease that was unrelated to the study drug. Study drug treatment was discontinued for three patients in each treatment arm due to SAEs and all patients recovered; no SAE leading to discontinuation was reported in more than one patient in either treatment arm.

The overall incidence of antidrug antibodies was 10.5% in the Eflapegrastim Arm and 4.4% in the Pegfilgrastim Arm and the incidence of anti‐PEG antibodies was 39.6% in the Eflapegrastim Arm and 64.9% in the Pegfilgrastim Arm. Treatment‐induced neutralizing antibodies were not detected for any patient in either treatment arm. In all cases, the presence of these antibodies was not associated with demonstrable effects on pharmacokinetics, safety, or efficacy.

## 4 DISCUSSION

This randomized Phase 3 study was the second study required for registration that compared the efficacy and safety of eflapegrastim to pegfilgrastim for reducing the risk of CIN in patients with Stage I‐IIIa ESBC. The study met all of the primary and secondary endpoints, demonstrating noninferior efficacy between eflapegrastim, at a lower G‐CSF dose (3.6 mg) than pegfilgrastim (6.0 mg G‐CSF), in reducing severe neutropenia and neutropenia‐related complications, including FN, which is associated with myelosuppressive TC therapy. Eflapegrastim and pegfilgrastim had comparable safety profiles that were consistent with what has been reported previously with pegfilgrastim. The noninferior efficacy and comparable safety results from this registration study support the results of another identically designed Phase 3 study (ADVANCE, NCT02643420) conducted in parallel, involving patients with ESBC undergoing TC therapy who were treated with eflapegrastim (n = 196) or pegfilgrastim (n = 210).^14^

In this study, differences in DSN, an objective measure based on ANC analyzed in an independent central laboratory and widely used in G‐CSF studies as a primary endpoint,^1^, ^2^ demonstrated the noninferiority of eflapegrastim to pegfilgrastim in Cycle 1, despite the lower dose (3.6 mg G‐CSF) of eflapegrastim compared to pegfilgrastim (6 mg G‐CSF); the noninferiority was maintained for the duration of treatment (*P* < .0001, all 4 cycles). No significant differences were observed between the Eflapegrastim Arm and the Pegfilgrastim Arm in any of the four cycles for time‐to‐ANC recovery, depth of ANC nadir, incidence of FN and neutropenic complications, and successful delivery of prescribed RDI.

Study drug‐related AEs occurred at a similar incidence with eflapegrastim (63%) and pegfilgrastim (61%), but the incidence of serious AEs was lower for eflapegrastim (10%) than pegfilgrastim (16%). Moreover, study drug–related serious AEs for the two arms were the same (2%) and the incidence of discontinuations due to study drug‐related AEs was also low (3% each). Overall, eflapegrastim was safe in patients receiving TC, and despite higher ANC values with eflapegrastim, eflapegrastim‐related AEs occurred at rates consistent with those previously reported for filgrastim and pegfilgrastim, including bone pain and other related musculoskeletal complaints.^12^, ^15^, ^16^, ^17^, ^18^, ^19^

Patients ≥65 years of age accounted for 37% of patients in the Eflapegrastim Arm and 34% of patients in the Pegfilgrastim Arm. This subset of patients is more susceptible to toxicity from chemotherapy regimens than patients <65 years of age,^20^, ^21^ and prophylactic use of G‐CSF is recommended.^22^ The results of this study show that eflapegrastim is efficacious and well‐tolerated in patients ≥65 years of age. The DSN with eflapegrastim was noninferior to pegfilgrastim and there were no notable differences in the types or incidence of AEs in patients treated with eflapegrastim or pegfilgrastim.

For all patients, the risk of CIN remains a significant concern for patients undergoing chemotherapy, as the condition frequently results in chemotherapy delays, dose reductions, and treatment discontinuations, all of which potentially compromise long‐term outcomes.^20^, ^23^, ^24^, ^25^, ^26^ Moreover, in a previously reported study involving community oncology patients undergoing chemotherapy, 10.7% of patients experienced FN, with most of these events (58.9%) occurring in the first cycle. In this same study, 10.9% of the breast cancer patients experienced FN and 21.3% experienced either FN or severe neutropenia despite prophylactic colony stimulating factor use.^27^ While the overall rates of FN are relatively low, especially with G‐CSF prophylaxis, patient consequences can be disproportionally unfavorable, resulting in neutropenic complications, costly hospitalization, the use of broad‐spectrum antibiotics, and increased mortality rates.^28^, ^29^

Since pegfilgrastim was initially approved in 2002, there has been a rapid development of innovative, effective cancer treatments, including numerous targeted and immunotherapy agents.^30^, ^31^ These novel drugs are used as monotherapy or in combination with standard chemotherapy, and have conferred increased survival benefits for oncology patients with early‐ or advanced‐stage disease.^30^, ^31^ However, the potential for these medications is limited by the development of CIN, which may significantly impede the completion of a patient's specified chemotherapy regimen.^24^, ^27^ The results from this study demonstrate noninferior efficacy and comparable safety for eflapegrastim at a lower G‐CSF dose vs pegfilgrastim. The potential for the increased potency of eflapegrastim to deliver improved clinical benefit warrants further clinical study.

## CONFLICT OF INTEREST

Gajanan Bhat, Shanta Chawla, and Steven Hasal are employees of Spectrum Pharmaceuticals, Inc.

## AUTHOR CONTRIBUTIONS

Patrick Wayne Cobb: investigation, data review, writing‐review, and editing. Yong Wha Moon: investigation and writing‐review and editing. Gajanan Bhat: conceptualization, methodology, data curation, formal analysis, and writing‐review and editing. Shanta Chawla: data curation and writing‐review and editing. Steven J. Hasal: data curation, visualization, and writing‐original draft. Klára Mezei: investigation and writing‐review and editing. István Láng: investigation and writing‐review and editing. Lee S. Schwartzberg: conceptualization, investigation, writing‐review, and editing.

## Data Availability

The data that support the findings of this study are available from the corresponding author upon reasonable request.
